# Cylindrical TGR as early radiological predictor of RLT progression in GEPNETs: a proof of concept

**DOI:** 10.1038/s41598-024-66668-9

**Published:** 2024-07-09

**Authors:** Federica Scalorbi, Enrico Matteo Garanzini, Giuseppina Calareso, Chiara Marzi, Gabriella Di Rocco, Giovanni Argiroffi, Michela Baccini, Sara Pusceddu, Alfonso Marchianò, Marco Maccauro

**Affiliations:** 1 Nuclear Medicine Department, Fondazione IRCCS Istituto Nazionale Dei Tumori, Milan, Italy; 2 Department of Radiodiagnostics and Radiotherapy, IRCCS Fondazione Istituto Nazionale Tumori, Milan, Italy; 3 Department of Statistics, Computer Science, Applications “G. Parenti”, University of Florence, Viale Morgagni 59, 50134 Florence, Italy; 4 Post-Graduation School of Radiology, Department of Health Sciences, University of Milan, Milan, Italy; 5 Department of Medical Oncology, Fondazione IRCCS Istituto Nazionale dei Tumori, Milan, Italy

**Keywords:** GEP-NETs, TGR, RLT, RECISTv1.1, Disease progression, Cancer, Neuroendocrine cancer, Medical imaging, Therapeutics

## Abstract

This study aims to assess the predictive capability of cylindrical Tumor Growth Rate (cTGR) in the prediction of early progression of well-differentiated gastro-entero-pancreatic tumours after Radio Ligand Therapy (RLT), compared to the conventional TGR. Fifty-eight patients were included and three CT scans per patient were collected at baseline, during RLT, and follow-up. RLT response, evaluated at follow-up according to RECIST 1.1, was calculated as a percentage variation of lesion diameters over time (continuous values) and as four different RECIST classes. TGR between baseline and interim CT was computed using both conventional (approximating lesion volume to a sphere) and cylindrical (called cTGR, approximating lesion volume to an elliptical cylinder) formulations. Receiver Operating Characteristic (ROC) curves were employed for Progressive Disease class prediction, revealing that cTGR outperformed conventional TGR (area under the ROC equal to 1.00 and 0.92, respectively). Multivariate analysis confirmed the superiority of cTGR in predicting continuous RLT response, with a higher coefficient for cTGR (1.56) compared to the conventional one (1.45). This study serves as a proof of concept, paving the way for future clinical trials to incorporate cTGR as a valuable tool for assessing RLT response.

## Introduction

Neuroendocrine neoplasms (NENs) are a group of tumours arising from the diffuse neuroendocrine system. Well-differentiated gastro-entero-pancreatic tumours (GEP-NETs) consist of carcinoids of the gastrointestinal tract and represent the most common NEN subtype. NET reported incidence is 3.56/100.000 cases per year, with an increasing trend in Western countries over the last decades^1–4^, principally due to the improved capability of imaging techniques to detect small lesions**.** Based on the World Health Organization (WHO) annual report 2019, GEP-NETs are classified as low-grade (G1), intermediate-grade (G2), and high-grade (G3), according to the proliferative index (MIB1 or Ki-67) that reflects the cellular biological aggressiveness^5,6^. In line with the Surveillance, Epidemiology and End Results (SEER), at the time of diagnosis, the whole NET population comprises localised disease in 53% cases, locoregional in 20%, and distant metastasis in 27% (from the Surveillance, Epidemiology End Results (SEER) database). These last mainly occur in the liver, although mesentery, peritoneum, and abdominal lymph nodes are frequently involved, particularly for small bowel disease^7^.

Surgery is the therapy cornerstone, being the only curative option. Unfortunately, many GEP-NETs are late-diagnosed and therefore are not amenable to curative treatments. In these cases, the systemic approach is mandatory, such as somatostatin analogues (SSA), targeted therapies, radioligand therapy (RLT) or chemotherapies^8^. RLT is based on a radioligand that targets SSA receptors expressed on well-differentiated GEP-NET cell surfaces. Among radiotracers, 177-Lu-DOTATATE is currently the most widely administered radiodrug, according to EMA and AIFA approval^9,10^, due to its low toxicity and positive effect in terms of survival, as confirmed by the NETTER-1 trial^11,12^.

Treatment response radiological assessment is based on lesion dimensional change during time, evaluated by contrast-enhanced (ce) Computer Tomography (CT) or Magnetic Resonance Imaging (MRI). Tumour size measurements are mainly performed following Response Evaluation Criteria in Solid Tumours version 1.1 (RECIST 1.1)^13^. Major diameters of target lesions are summed to obtain a “sum of lesion diameters'' (SOD), the percentage variation of which during time categorises four possible responses to treatment: complete response (CR), partial response (PR), stable disease (SD) or progressive disease (PD)^14^. Treatment response assessment based just on tumour size changes is associated with several pitfalls since GEP-NETs tend to stabilise or initially increase in size even when responding to the treatment^15,16^. Additionally, central tumour necrosis is a frequently reported event during RLT. This aspect can be challenging to assess with radiological criteria due to the ‘false-positive’ increase in lesion diameters and the decrease in concomitant density. Moreover, tumour shrinkage is known to be a late event**;** indeed, most patients treated with RLT achieve SD rather than PR, observed in only 18–30% of cases. It is reported that PD patients have similar overall survival (OS) as SD ones due to an underestimation of RLT response applying RECIST 1.1. Conversely, tumour growth increase may be delayed in slow-growing GEP-NETs that can be wrongly classified as SD^17–19^. Therefore, RECIST1.1 presents multiple constraints, and their application on slow-growing diseases must be carefully considered.

To overcome RECIST 1.1 limitations, alternative radiological criteria have been proposed, such as Choi, which combines size and density variation on ceCT^20^. Several studies have compared the two criteria on NET evaluation, reporting equal or significantly better results for Choi^21–24^. NETs are typically evaluated on arterial phase due to their intense contrast enhancement^25^, while Choi are outlined on the venous portal one, leading to a limited application in clinical practice. Moreover, both RECIST and Choi, estimated at the end of the therapy, supply a static view of the disease without predicting tumour change over time. These observations outline the need for new tools to accurately evaluate GEP-NETs' prognosis, particularly during a systemic treatment such as RLT.

Tumour growth rate (TGR) provides quantitative and dynamic information about tumour response, quantifying volume change over time^26^. TGR appears to be simpler to use and more reproducible than other new tools that imply total volume contouring. Furthermore, previous studies have shown that TGR was correlated with treatment response and clinical outcomes^27,28^, even for GEP-NETs^29–35^. The estimation of the TGR is based on the assumption of lesion spherical shape^36^, which is a significant limitation of TGR application in clinical practice due to the frequent evidence of irregularly shaped lesions. Thus, tumour volume assessment with classical TGR formula does not reflect actual shape, giving underestimation or overestimation of volume change over time. Our study proposes an alternative and more accurate method to calculate TGR, from now on named cylindrical TGR (cTGR). The aim of the study is to evaluate the accuracy of cTGR, compared with the conventional TGR, as predictors of RLT response to select in advance non-responder patients.

## Methods

### Participants

Data from GE-PNET patients treated with RLT through the Foundation IRCCS, Istituto Nazionale Tumori—Milan—Italy, from May 2019 to December 2021 were retrospectively collected. All the patients received 177Lu-Oxodotreotide (Lutathera®) and SSA, in accordance with EMA and AIFA indications^9,10^. Before RLT, tumour burden and SSA expression were assessed by ceCT/MRI scan and 68 Ga-DOTA-SSA PET/Octreoscan, respectively. For each patient the following clinical data were collected: age, gender, WHO grading, primary tumour (categorised as midgut, foregut, hindgut), Eastern Cooperative Oncology Group (ECOG) performance status (ECOG-PS, categorised as 0 or 1–2) and previous lines of systemic treatments (categorised as 1 or ≥ 2). Inclusion criteria were: age ≥ 18 years, histopathological diagnosis of G1 or G2 WHO 2019 (data extracted from pathological reports), disease progression after at least one previous line of treatment, three contrast enhanced ceCT/MRI scans per patient. Exclusion criteria were: absence of primary tumour diagnosis, Ki67 assessment, radiological/nuclear medicine imaging evaluation, signed informed consent.

This observational study was approved by the Ethical Committee of National Institute for Tumor of Milan (Study number: INT 6/20) and was conducted in accordance with the Principles of Declaration of Helsinki (1964). All the patients have given written informed consent for the participation in the study.

### CT/MRI examination

After receiving informed consent, ceCT scans were acquired under baseline conditions and after intravenous administration of iodinated contrast medium with arterial and venous phase scan. Occasionally, the studies were integrated with late or urographic phases according to diagnostic/clinical needs. In most cases, the portal phase was favoured for measuring target lesions (usually liver metastasis or abdominal nodes). Contrast enhanced MRI was used when CT was not available or when it was clinically preferable. A ceCT scan was performed at baseline (within 3 months from the start of RLT), one ad interim (between the second and the third RLT cycle), and the last ceCT was acquired two months after the completion of RLT (referred to as follow-up CT). To avoid bias, target lesion comparison over time was performed using the same scan timing. All the images were retrospectively reviewed by two expert dedicated radiologists, involved in the multidisciplinary management of NETs. Disagreements were resolved by a third independent radiologist without intra-observer agreement evaluation.

**Figure 1:**
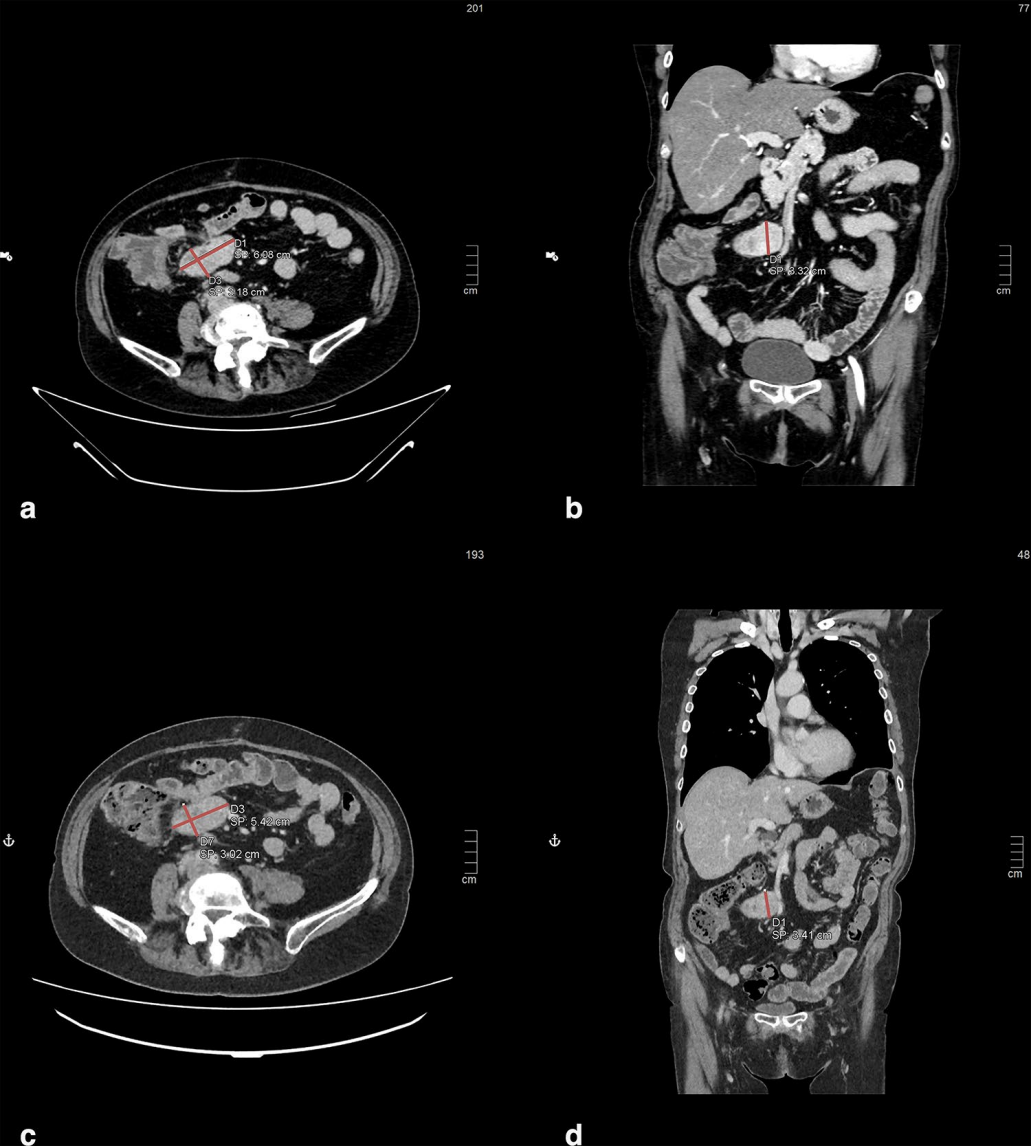
Example of three-axes assessment of a GEP-NET lesion approximated to an elliptic cylinder on a ceCT scan acquired during the portal phase (axial (**a** and **c**) and coronal (**b** and **d**) views). Panels (**a**) and (**b**) represent the baseline scans, while panels (**c**) and (**d**) include the interim image. If the lesion's shape were modeled as a sphere, its diameter would correspond to the major axis delineated in the axial plane (baseline (**a**) and interim (**c**)).

**Figure 2:**
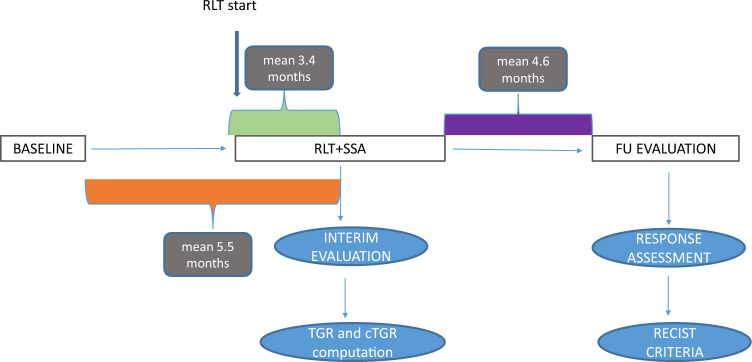
Study design. Interim evaluation was performed after 2 RLT administrations.

At both the baseline and interim examinations, expert radiologists identified each target lesion and provided an approximate description, characterizing it as either a sphere or an elliptical cylinder. For spherical approximation radiologists measured the diameter in the axial view. Conversely, when lesions were approximated as elliptical cylinders, first of all the radiologist determined the orientation of each target lesion and chose planes on which to measure the axes of the elliptical base and the height. Specifically, with no exception, in our study radiologists systematically determined a vertical development of lesions, so that they estimated the lengths of the axes of the elliptical base in the axial plane and the height of the cylinder in the coronal/sagittal views (an example of axes delineation and height of a single lesion is depicted in Fig. 1). Once the planes were defined, all measurements were taken considering the slice showing the largest diameter in the defined plane, following RECIST 1.1 criteria^13^. The updated RECIST criteria version 1.1, indeed, allow to take measurements in planes other than the acquisition plane when working with isotropic images, as in our case. Therapy response was quantified (comparing baseline and follow-up scan) and was binary categorised (progression or no-progression) in accordance with RECIST1.1. Non-target lesions were not considered in the TGR evaluation, as described by previous publications^18,33,36^. Study design is illustrated in Fig. 2.

### TGR computation

Classical TGR was assessed in accordance with Collins’ formula^36^, i.e., each lesion was considered spherical, and its diameter was measured in the axial view.

To calculate the cTGR, we estimated the volume of each lesion, considering it as an elliptical cylinder, i.e., a cylinder with an elliptical base (Eq. 1):

1 $$V=\pi abh$$

where $a$ and $b$ are the major and minor radii of the elliptical base in the axial plane, respectively, and $h$ is the elliptic cylinder height in the coronal view (example in Fig. 1).

The choice to consider this formula was related to the direct observation of the real shape of GEP-NET lesions on CT scan in clinical practice. In detail, considering the axial view we noticed that the major and the minor axes are frequently quite diverse so the spherical formula may not include this feature. Furthermore, in the coronal view the height of the lesion could be a further measurement to consider that differs from the previously mentioned axes. Indeed, to correctly estimate the lesion volume these three measurements must be borne in mind in the mathematical formula. Hence, we suggest considering a cylinder with an elliptical base as the most appropriate geometric shape for characterizing the lesion. This entails a slightly extended evaluation time for the radiologist, without impacting either the acquisition duration or the patient's participation. To numerically confirm the nonsphericity of the identified lesions, we computed the sphericity index, which consists of the ratio of the surface area of a sphere occupying the same volume as the object under examination to the surface area of the object itself^37^ (Eq. 2).

2 $$\Psi =\frac{\sqrt[3]{36\pi {V}^{2}}}{A}$$

where $V$ is the volume, estimated using Eq. (1) ^37^, and $A$ is the surface area of the lesion, ie., $A\cong 2\pi ab+2\pi h\sqrt{\frac{{a}^{2}+ {b}^{2}}{2}}$

### Statistical analysis

We conducted the same statistical analyses on the whole sample and on a subset of lesions exhibiting the largest discrepancy between conventional and cylindrical TGR values, where $|TGR-cTGR|> 2$, to examine the effectiveness of introducing cTGR in lesions undergoing morphological changes that affect not only volume but also shape during the longitudinal follow-up.

We explored the capabilities of the cTGR to predict early disease progression after only two RLT administrations through two complementary statistical analyses (similarly applied for conventional TGR). First, we analysed TGR’s ability to discriminate the class of progression through the Area Under the Receiver Operating Characteristic (AUROC) computation. We compared the AUROC values obtained using either the cTGR or the conventional TGR as predictors of progression class—the higher the AUROC value, the better the model is at distinguishing between different classes of progression.

Then, we modelled the relationship between the change in TGR (conventional or cylindrical) and the response to RLT (expressed as the percentage change in the sum of the major diameters of baseline and follow-up lesions), specifying two separate linear regression models, one including conventional TGR and one including cTGR, together with the following covariates: age, gender, primary tumour, ECOG-PS, lines of treatment, WHO grading. Both AIC and BIC^38^ were computed in order to compare the performance of the two models (dealing with the trade-off between the goodness of fit of the model and the simplicity of the model). The one with the smaller AIC (or BIC) indicates a better-fitting model than the one with the larger AIC (or BIC)^39^.

## Results

Table 1 shows the clinical characteristics of the study population. In detail, 58 patients were included, 34 of whom were female (58.62%). All patients had liver metastases and, in the majority of cases, had abdominal lymph node metastases too. No metastases were present in the bones, lungs or mediastinal lymph nodes. One patient had metastases involving the gynecological system. In accordance with RECIST 1.1, eleven (19%) were classified as PR, 42 (72.4%) as SD, and 5 (8.6%) as PD after RLT. Mean time between baseline CT scan and start of RLT was 5.5 months, whereas between the end of treatment and follow-up CT 4.6 months.

**Table 1:** Study population (n = 58) clinical and radiological features.

Category	Data	Values
Study population	Total number	58
	Females (%)	34 (58.6%)
	Males (%)	24 (41.4%)
Age (years)	Mean [min, max]	62.8 [27, 82]
Primary tumour	Midgut (%)	34 (58.6%)
	Foregut (%)	20 (34.4%)
	UO (unknown origin) (%)	4 (7%)
Line of therapies	2nd line (%)	42 (72.4%)
	3rd or further line (%)	16 (27.6%)
ECOG-PS	ECOG 0 (%)	50 (86.2%)
	ECOG 1–2 (%)	8 (13.8%)
WHO grading	G1 (%)	26 (44.8%)
	G2 (%)	32 (55.2%)
4 RLT administrations	Total (%)	56 (96.5%)
RLT response	PR (partial response) (%)	11 (19%)
	SD (stable disease) (%)	42 (72.4%)
	PD (progressive disease) (%)	5 (8.6%)
RLT continuous response	Mean [min, max]	− 9.78 [ − 65, 37.74]
Conventional TGR	Mean (standard deviation)	− 2.43 (8.77)
Cylindrical TGR	Mean (standard deviation)	− 2.92 (5.89)

*ECOG-PS*: ECOG performance status; *PD*: progressive disease; *PR*: partial response; *RLT*: radioligand therapy; *SD*: stable disease; *UO*: unknown primary GEP-NET tumour.

**Figure 3:**
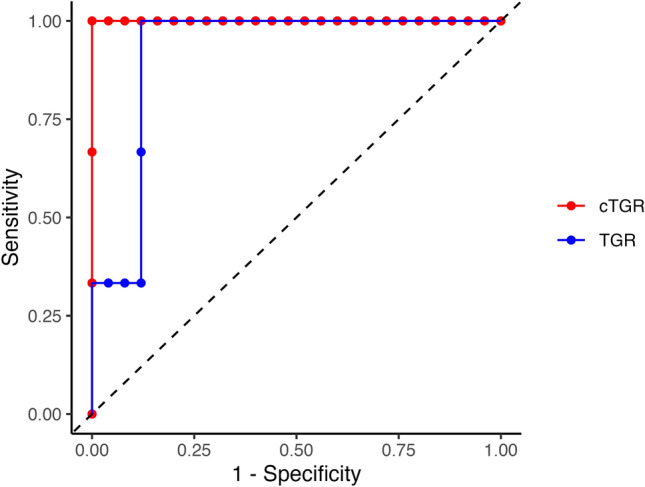
ROC curves for conventional TGR and cTGR in the subset of lesions exhibiting the largest discrepancy between conventional and cylindrical TGR values. The black dashed line represents the chance level.

Our findings support the utility of TGR, particularly in its cylindrical formulation, to predict PD class and changes in SOD. Specifically, in the analysis carried out on the subset of lesions exhibiting the largest discrepancy between conventional and cylindrical TGR values, the Area Under the Receiver Operating Characteristic (ROC) curve (AUROC) for cTGR was 1 (95% CI [1, 1]), while the AUROC for conventional TGR was 0.92 (95% CI [0.8, 1]) (Fig. 3 and Table 2). The combination of sensitivity and specificity leading to the highest Youden’s index^40^ corresponded to a cut-off threshold of 9.59 for the cTGR and 5.84 for the TGR (details in Table 2). Applying the cut-offs mentioned above, both cTGR and TGR obtained a sensitivity (i.e., the ability to identify the non-responders correctly) equal to 100%. At the same time, the cTGR achieved a higher specificity (i.e., the ability to identify the responder patients correctly) than the conventional TGR (100% and 88%, respectively).

**Table 2:** Performance of the prediction of the PD class for conventional and cylindrical TGR.

	TGR	cTGR
Subsample exhibiting the largest discrepancy between conventional and cylindrical TGR values		
AUROC	0.92 [0.80, 1.00]	1.00 [1.00, 1.00]
Youden’s index	0.88	1.00
Sensitivity	1.00	1.00
Specificity	0.88	1.00
Cut-off threshold	5.84	9.59
Whole sample		
AUROC	0.79 [0.47, 1.00]	0.82 [0.49, 1.00]
Youden’s index	0.73	0.74
Sensitivity	0.80	0.80
Specificity	0.92	0.94
Cut-off threshold	5.84	5.85

*AUROC*: area under the ROC curve; *cTGR*: cylindrical TGR; *ROC*: receiver operating characteristic; *TGR*: tumor growth rate.

Additionally, when predicting the continuous SOD change, the model incorporating cTGR slightly outperformed the model including conventional TGR as one of its predictors. This is evident from the lower Akaike Information Criterion (AIC) (214.59 vs. 221.13), lower Bayesian Information Criterion (BIC) (224.96 vs. 231.50), and slightly higher coefficient (1.56 vs. 1.45) associated with cTGR compared to conventional TGR (details in Supplementary Table S1 online).

**Figure 4:**
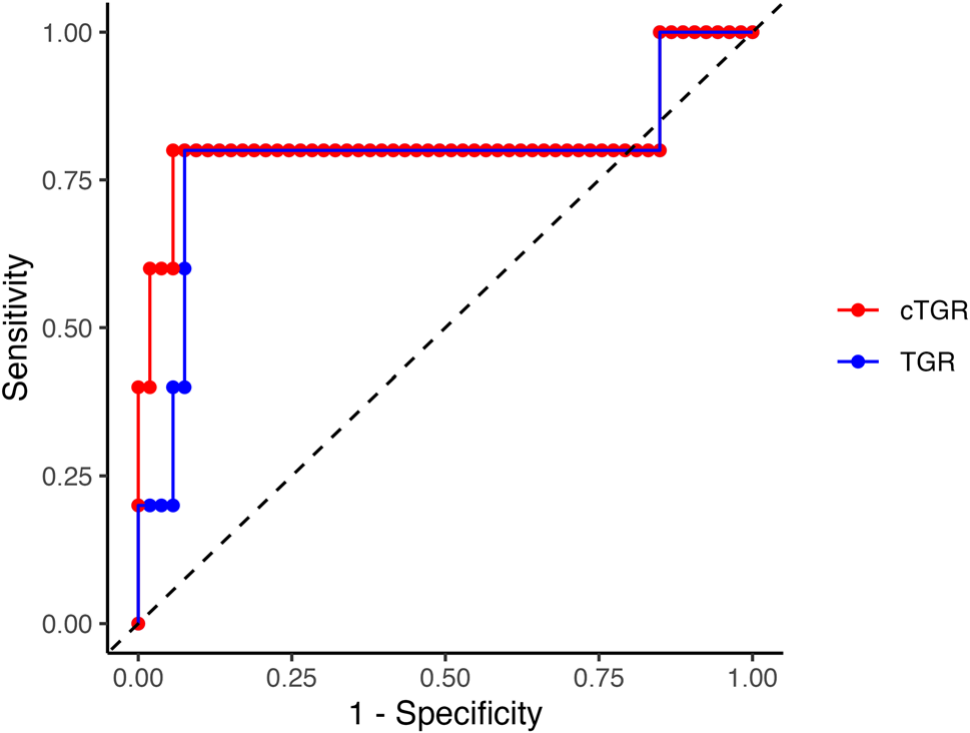
ROC curves for conventional TGR and cTGR in the whole sample. The black dashed line represents the chance level.

The ability of the TGRs to early discriminate against the PD class was confirmed also in the whole sample. Specifically, the AUROC for cTGR was 0.82 (95% CI [0.49, 1]), while the AUROC for conventional TGR was 0.79 (95% CI [0.47, 1]) (Fig. 4 and Table 2). The regression model that incorporated cTGR and the one involving conventional TGR produced highly comparable outcomes in forecasting the continuous change in SOD. These results closely resembled those achieved within the subset of lesions where there was a notable difference between conventional and cylindrical TGR values (details in Supplementary Table S2 online).

**Figure 5:**
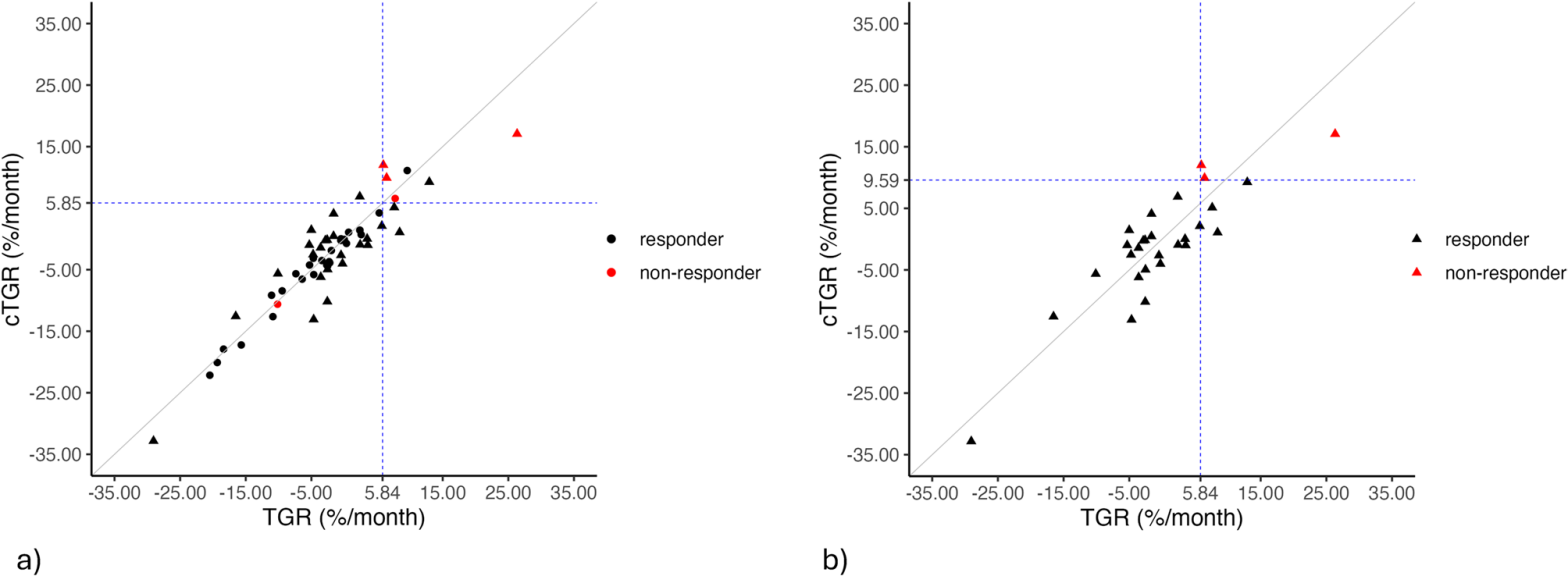
Scatterplot illustrating the relationship between TGR and cTGR for the whole sample (**a**) and the subset of lesions exhibiting the largest discrepancy between TGR and cTGR values (**b**), where the points belonging to the subset of lesions exhibiting the largest discrepancy between TGR and cTGR values have been represented as triangles. The gray line represents the bisector line, providing an indication of where the points would lie if TGR and cTGR were equal.

We graphically reported the relationship between TGR and cTGR in Fig. 5. As expected, the two measures are linearly and positively correlated, but the TGR values are different depending on the geometrical approximation of the lesion shape (the red dashed bisector in Fig. 5 provides an indication of where the points would lie if TGR and cTGR were equal).

The slight difference in the reported AUROC values may be due the fact that RLT response and TGR are highly correlated, as both are based on a spherical lesion model, unlike cTGR. Furthermore, in the case of a study population composed of both spherical and cylindrical lesions the difference in the accuracy between cTGR and TGR may be diminished. For this reason, we suggest a preliminary evaluation of lesion shape on both axial and coronal view to consider which of the two TGR formulas would provide the greater accuracy in the volume estimation. This can partially explain the unexpected small difference between cTGR and TGR in the predictive power of disease progression.

## Discussion

To our knowledge this is the first study that presents a compelling approach challenging the conventional consideration of spherical lesions calculating TGR through application of a modified formula, compared to the classical one proposed by Collins^36^. Specifically, we proposed to characterise the lesions as elliptical-based cylinders, enabling a closer approximation to the true lesion morphology. This innovative approach offers several distinct advantages. Firstly, it eliminates the need for laborious manual segmentation, saving valuable time and resources. Secondly, by capturing the specific elliptical shape, we can derive more accurate measurements of both volume and surface area, which are crucial factors in calculating the TGR and the SOD values. Furthermore, by relaxing the assumption of sphericity, our methodology may effectively capture subtle morphological changes that often go unnoticed when lesions are solely regarded as spherical. While our results should be interpreted descriptively, the subtle differences observed in AUROC values, in the linear regression model coefficients, as well as AIC and BIC values, suggest that the application of the cTGR formula model migth be preferred. This is particularly true when the lesions undergo changes in shape, not just volume, during the longitudinal follow-up, as demonstrated in the analysis conducted on the subset of lesions exhibiting the largest discrepancy between conventional and cylindrical TGR values. Using ROC curves, we also assessed the optimal predictive cut-off values for TGR and cTGR, calculated at interim CT scan.

There is a small number of studies that have investigated the role of conventional TGR in the prediction of RLT response. In detail, Pettersson et al. have investigated the power of TGR in predicting RLT response in pancreatic NETs (pNETs) only, reporting that patients with TGR $\ge$ 0.5%/month at 10 months from baseline had shorter median PFS (Hazard Ratio 2.01)^34^. TGR was also used to predict RLT response by Prasad et al.^41^. They calculated TGR before RLT to predict objective response rate (ORR) at the end of treatment (optimal TGR cut-off < 1.18%/month, AUROC = 0.75) and through 12-month follow-up (TGR < 0.33%/month, AUROC = 0.82), respectively. We reported higher TGR cut-offs, probably due to the differences in the study population (GEP-NETs vs pNETs only), time points at which TGRs were assessed and different outcomes (progression vs ORR). Lamarca et al. evaluated the value of TGR3 (calculated 3 months before treatment choice) in the PFS prediction in GREPONET study (systemic therapies vs “watch and wait” strategy in GEP-NETs)^31^. They reported that TGR 0.8%/month was the optimal cut-off to predict PFS (26.3 vs 9.3 months). A further publication by Lamarca et al.^32^, based on GREPONET study, confirmed that TGR3 > 0.8 was an independent prognostic factor for PFS (HR 2.2). Although in our study both the TGRs were calculated 3.4 months from RLT start, our reported cut-offs are not comparable with those of Lamarca, probably due to the features of the study population. Indeed, GREPONET’s patients were subjected to different systemic treatments (RLT in 28% of cases) while our study was targeted on RLT response only. Conventional TGR has been applied to predict response to treatment and prognosis in GEP-NETs treated with Lanreotide vs. placebo (CLARINET trial)^42^. Indeed, Dromain et al. reported that TGR3 is a robust predictor of PFS and that high TGR value (10%/month), calculated 12 weeks after randomisation, was an early predictor of worse PFS (HR 8)^30^. This cut-off is not in line with our results regarding TGR values (5.84%/month) in the whole population. This conflicting result can be explained by considering the different treatment that the patients in Dromain's study underwent (any line of systemic therapies) compared to the radiometabolic treatment that is the subject of this study. However, our TGR value is consistent with the cut-off reported in Dromain et al.^33^ on the use of pre-treatment TGR as an independent predictor of PD or death. This result underscores the clinical utility of early TGR measurement in predicting disease progression as a kind of biomarker of proliferative activity. Specifically, the application of TGR0 revealed that a proportion of tumors were actively growing during the pre-treatment period, despite being classified as SD according to RECIST v1. In details, using a TGR0 cut-off of 4%/month, patients with TGR0 > 4%/month had a four-fold higher risk of PD/death compared to those with TGR0 ≤ 4%/month. The utility of applying TGR before treatment, or ad interim as in our study, would therefore allow for the early selection of patients with a rapid growth rate, who consequently show a significantly increased risk of progressing after treatment.

Recently Wang et al.^35^ have highlighted the value of TGR0, reporting that > 11.7%/month was related to short time to first therapy (HR 2.4) and OS (HR 6.57). Moreover, multivariate analysis highlighted that TGR0, but not baseline Ki67, was predictive of grading increase during time. We also performed a multivariate analysis, finding that Ki67 is not a predictor of progression after RLT, similarly to Dromain^33^ and Wang^35^, in contrast with Lamarca^32^, and Pettersson^34^. These discrepancies must be investigated by further independent studies, since Ki67 is one of the most applied biomarkers to stratify GEP-NET aggressiveness and, therefore, to plan GEP-NET decision making. The above-mentioned publications have highlighted the importance of implementing RECIST criteria to increase CT/MRI diagnostic accuracy in predicting GEP-NET response to treatment.

We acknowledge several limitations in our study. First, the study was carried out on a small cohort of patients, therefore the reported cTGR and TGR cut-off values must be confirmed by an external validation. Second, this study did not assess the intra- and inter-rater variability of cTGR measurements. Future studies involving a larger patient cohort should address this aspect to ensure the robustness and reliability of the cTGR measurement method. Third, we are aware of the absence of a pre-treatment cTGR evaluation and the relatively short follow-up period. Lastly, RLT response and TGR are highly correlated, as both are based on a spherical lesion model, unlike cTGR.

Notwithstanding the reported constraints, this study can be considered a proof of concept to focus the radiologist’s attention on the emerging role of TGR and in particular on cTGR. The accurate estimation of lesion volume change over time can be of great importance in clinical practice, in order to overcome RECIST pitfalls, in particular in slow growing tumours treated with systemic treatment, such as RLT. In this setting of patients, an early and non-invasive evaluation is pivotal in order to select non-responder patients that will not reach a net benefit from further radiation exposure. Furthermore, cTGR application could also limit RLT related toxicities and sort out in advance patients that can benefit from alternative therapies.

### Supplementary Information

Supplementary Tables.

## Data Availability

The datasets generated during and/or analysed during the current study are available from the corresponding author on reasonable request.
