# Supplementary material for: Cylindrical TGR as early radiological predictor of RLT progression in GEPNETs: a proof of concept
Source: Sci Rep. 2024 Jul 9;14:15782. doi: 10.1038/s41598-024-66668-9 (PMC11233714; doi:10.1038/s41598-024-66668-9)
Supplement: Supplementary file 1 — Supplementary Tables. [file 41598_2024_66668_MOESM1_ESM.pdf]

Supplementary Materials for the manuscript entitled "**Cylindrical TGR as Early Radiological Predictor of RLT Progression in GEPNETs. A Proof of Concept**"

Federica Scalorbi[1,^], Enrico Matteo Garanzini[2,^], Giuseppina Calareso[2], Chiara Marzi[3,*], Gabriella Di Rocco[2,4], Giovanni Argiroffi[1], Michela Baccini[3] , Sara Pusceddu[6], Alfonso Marchianò[2], Marco Maccauro[1]

[1]Nuclear Medicine Department, Fondazione IRCCS Istituto Nazionale dei Tumori, Milan, Italy
[2]Department of Radiodiagnostics and Radiotherapy, IRCCS Fondazione Istituto Nazionale Tumori, Milan, Italy.
[3]Department of Statistics, Computer Science, Applications "G. Parenti", University of Florence, Florence, Italy
[4]Post-graduation School of Radiology, Department of Health Sciences, University of Milan, Milan, Italy.
[6]Department of Medical Oncology, Fondazione IRCCS Istituto Nazionale dei Tumori, Milan, Italy

[^]Federica Scalorbi and Enrico Matteo Garanzini contributed equally to this work.
*Corresponding Author: Chiara Marzi, chiara.marzi@unifi.it. Department of Statistics, Computer Science, Applications "G. Parenti", University of Florence. Viale Morgagni 59, 50134, Florence, Italy.

Supplementary Table S1: Linear regression in a subset of lesions exhibiting the largest discrepancy between TGR and cTGR

**Linear regression with cTGR**				
	Coefficient	SE	95% CI	p-value
cTGR	1.56	0.28	0.97 – 2.14	0.000
Gender	-3.33	4.62	-13.01 – 6.35	0.48
M0F1	-4.83	6.70	-18.86 – 9.20	0.48
Age	-0.65	0.25	-1.18 – -0.11	0.02
Lines	-1.59	4.98	-12.02 – 8.84	0.75
ECOG	15.02	9.02	-3.85 – 33.90	0.11
Grading	-1.89	4.92	-12.19 – 8.40	0.70
AIC	214.59			
BIC	224.96			
**Linear regression with TGR**				
	Coefficient	SE	95% CI	p-value
TGR	1.45	0.32	0.78 – 2.13	0.000
Gender	-2.37	5.24	-13.35 – 8.61	0.66
M0F1	-3.65	7.73	-19.82 – 12.52	0.64
Age	-0.53	0.28	-1.12 – 0.06	0.07
Lines	-0.55	5.61	-12.30 – 11.20	0.92
ECOG	6.64	9.90	-14.10 – 27.38	0.51
Grading	3.53	5.80	-8.60 – 15.66	0.55
AIC	221.13			
BIC	231.50			

AIC: Akaike Information Criterion, BIC: Bayesian Information Criterion, CI: confidence interval, SE: standard error, TGR: tumour growth rate

Supplementary Table S2: Linear multivariate regression analyses performed in the whole population

**Linear regression with cTGR**				
	Coefficient	SE	95% CI	p-value
cTGR	1.17	0.25	0.66 – 1.67	< 0.001
Gender	-2.98	4.48	-12.00 – 6.05	0.51
M0F1	-9.60	5.06	-19.79 – 0.59	0.06
Age	-0.52	0.21	-0.94 - -0.10	0.02
Lines	0.93	3.54	-6.20 – 8.06	0.79
ECOG	3.48	6.87	-10.35 – 17.31	0.62
Grading	-3.45	4.58	-12.67 – 5.77	0.46
AIC	466.04			
BIC	486.11			
**Linear regression with TGR**				
	Coefficient	SE	95% CI	p-value
TGR	1.12	0.25	0.61 – 1.62	< 0.001
Gender	-3.12	4.54	-12.27 – 6.02	0.50
M0F1	-8.64	5.18	19.06 – 1.78	0.102
Age	-0.49	0.21	-0.91 – 0.06	0.03
Lines	0.99	3.58	-6.22 – 8.20	0.78
ECOG	0.71	6.83	-13.03 – 14.48	0.92
Grading	-1.56	4.65	-10.91 – 7.80	0.74
AIC	467.30			
BIC	487.37			

AIC: Akaike Information Criterion, BIC: Bayesian Information Criterion, CI: confidence interval, SE: standard error, TGR: tumour growth rate
